# Examining the influence of alcohol consumption on the protective function of social support in reducing intimate partner violence among adolescent girls and young women

**DOI:** 10.1080/17450128.2026.2702907

**Published:** 2026-07-15

**Authors:** Victoria Oyekunle, Andrew Gibbs, Kim Jonas, Andrew Tomita

**Affiliations:** a School of Health Systems and Public Health, University of Pretoria, Pretoria, South Africa; b Department of Psychology, University of Exeter, Exeter, UK; c Centre for Rural Health, School of Medicine, University of KwaZulu-Natal, Durban, South Africa; d Gender and Health Research Unit, South African Medical Research Council, Pretoria, South Africa; e Health Systems Research Unit, South African Medical Research Council, Pretoria, South Africa; f KwaZulu-Natal Research Innovation and Sequencing Platform (KRISP), College of Health Sciences, University of KwaZulu-Natal, Durban, South Africa

**Keywords:** Intimate partner violence, adolescent girls and young women, social support, alcohol use, South Africa

## Abstract

Intimate partner violence (IPV) remains a major public health and human rights concern in South Africa, particularly among adolescent girls and young women (AGYW). While social support is widely recognized as a protective factor against IPV, its effectiveness may be compromised in contexts of alcohol use. This study investigated the influence of alcohol use on the risk of IPV and the moderating role of perceived social support in reducing this risk. This study used baseline data from the HERStory 1 study, a cross-sectional survey of 4,399 adolescent girls and young women aged 15–24 years. Social support was measured using the Multidimensional Scale of Perceived Social Support (MSPSS). Multivariable logistic regression assessed main and interaction effects, with alcohol use as a moderator. Marginal effects and plots illustrated predicted IPV probabilities across social support and alcohol use levels. Emotional IPV (23.1%) was most prevalent, followed by physical (17.0%) and sexual (6.3%) IPV; 25.2% reported harmful alcohol use. Higher perceived social support was associated with reduced odds of emotional, physical, and sexual IPV across all support domains. Family and friends’ support showed strong protective associations. For example, family support was associated with lower odds of emotional (aOR = 0.53, 95% CI: 0.42–0.66), physical (aOR = 0.59, 95% CI: 0.45–0.75), and sexual IPV (aOR = 0.41, 95% CI: 0.29–0.58). In contrast, support from a significant other was not significantly associated with sexual IPV. Harmful alcohol use increased the odds of all IPV forms and attenuated the protective effects of social support. Additional age-stratified analyses indicated that social support was more consistently protective against IPV among adolescents (15–19 years) than young adults (20–24 years). Social support, particularly from family and friends, plays an important role in reducing IPV risk among young women, but its protective effect is diminished when alcohol use is present. These findings highlight the need for integrated IPV prevention strategies that strengthen external support networks and also address harmful alcohol use.

## Introduction

Nearly one in three women report to have experienced physical, emotional or sexual abuse at the hands of an intimate partner globally (World Health Organization [WHO], 2021). This alarming statistic underscores the widespread nature of intimate partner violence (IPV) and makes its prevalence among adolescent girls and young women (AGYW) particularly disturbing (Stöckl et al., 2014). AGYW often face unique vulnerabilities, such as limited economic independence, social stigma and unequal power dynamics in relationships, which can increase both their risk of experiencing IPV and the severity of its consequences (Sardinha et al., 2022; Wado et al., 2021).

IPV can have devastating impacts on young women going through important life phases, which can interfere with their education, lower their access to economic prospects, and cause long-term physical and mental trauma (Hing et al., 2021). The distinct historical, social, gendered and economic circumstances of South Africa make AGYW more at risk of experiencing IPV (Pakhomova et al., 2021). Widespread and sustained gender inequitable attitudes and practices that condone or accept abuse frequently contribute to the persistence of gender inequity (Chadambuka & Warria, 2019). In addition, due to young women’s relative lack of financial freedom, many may become dependent on others, keeping them in violent relationships (Conner, 2013). The issue is exacerbated by limited access to legal, medical and educational institutions, which leaves AGYW with few choices for recovery or escape paths. The stigma associated with IPV worsens these difficulties by discouraging survivors from coming forward or asking for assistance, thereby perpetuating cycles of isolation and silence (Camp, 2022; Sharafudeen, 2024).

Despite these complexities, social support has become a source of hope, an important shield that may minimize the effects of IPV and enable survivors to start over (Ogbe et al., 2020). Social support includes material, emotional and spiritual help from peers, family, friends, institutions and societal organizations. It can help to reduce feelings of loneliness, increase self-confidence, and make resources more accessible, such as legal guidance, psychological treatment and shelter (Machisa et al., 2018; Richardson et al., 2022). Strong social networks can provide an informal safety net in situations when systemic responses to IPV are frequently insufficient, thereby assisting AGYW in overcoming the difficulties of ending abusive relationships, and regaining their feeling of control and liberty.

While social support clearly has the ability to prevent IPV, little is known about how it works in South Africa, specifically regarding teenage girls and young women, with research and? initiatives having focused on structural or legal solutions (Ryan et al., 2018; Sere et al., 2021; Tshoane et al., 2023). For example, national action plans to stop violence against women, raise public awareness and make it easier for victims to get legal help have all been popular areas of focus, rarely considering the complex ways that community and personal connections might act as protective barriers against the damaging effects of IPV. Access to effective support is also sometimes hampered by obstacles such as cultural shame, fear of being judged and disjointed community structures, underscoring the necessity of focused efforts to maintain and grow these networks (Camp, 2022; Thaggard and Montayre, 2019).

Social support has been generally acknowledged as a protective factor against IPV as it gives people the practical and psychological resources needed to resist or leave abusive situations (Howell et al., 2018). According to studies conducted in sub-Saharan Africa, robust social networks, especially those derived from peers and family, can lessen exposure to IPV by promoting resilience and improving access to support services (Aboagye et al., 2023; Davies et al., 2023). However, harmful alcohol drinking by the victim may make the protective function of social support more difficult to implement and therefore less effective. Alcohol, due to how it can exacerbate conflict and impair judgment in relationships, is a well-established risk factor for women’s experience of IPV (Howell et al., 2018). It can decrease inhibition and escalate tensions, thereby contributing to the likelihood of violence. At the same time, alcohol use may also be an outcome of IPV, as women, including AGYW, may turn to drinking as a way to cope with trauma or manage symptoms of poor mental health resulting from abuse. Alcohol consumption can also reduce AGYW’s ability to perceive risk or regulate their circumstances, which may negate the protective effects of social support (Aboagye et al., 2023; Howell et al., 2018).

Understanding the relationship between social support and alcohol use is essential for creating targeted interventions that reduces IPV risks among AGYW in South Africa. This study aims to shed light on how alcohol consumption by the victims affects the protective function of social support in reducing intimate partner violence (IPV) among young women in South Africa. Specifically, it examines if and how different sources of social support (family, friends and significant others) moderate the association between alcohol use and the risk of experiencing emotional, physical or sexual IPV. To guide this inquiry, the study addresses the following research questions (WHO, 2021): How does alcohol use by the victim affect the likelihood of experiencing IPV? (Stöckl et al., 2014) Does perceived social support reduce the risk of IPV associated with alcohol use by the victim? (Sardinha et al., 2022) Do different sources of social support (family, friends, significant others) vary in their protective effects? Social support remains an essential yet underutilized component of IPV prevention and response strategies. Strengthening these networks not only addresses the immediate needs of survivors, but also lays the foundation for ensuring long-term societal change, and promoting resilience, equity and safety for South Africa’s young women.

## Materials and methods

### Study design

The HERStory 1 study was a stratified, population-based cross-sectional survey designed to estimate HIV incidence among adolescent girls and young women (AGYW) aged 15–24 years living in 10 South African subdistricts selected for the implementation of a combination HIV prevention intervention, funded by the Global Fund. Due to logistical constraints, data was ultimately collected from six of the 10 subdistricts between 2018 and 2019. Sampling was based on the 2011 Census Small Area Layers (SALs), and updated with more recent population estimates. A simple random sample of SALs was drawn within each district, and within these, 35% of households were systematically sampled. All AGYW aged 15–24 in selected households were invited to participate, yielding an overall weighted sample of approximately 7,300 participants after visiting an estimated 46,000 households. Data collection involved electronic questionnaires (Mobenzi Researcher), with the sensitive sections being self-completed by participants. Full details on the study design, sampling and data collection are available in the main HERStory survey report (Mathews et al., 2020).

For the present secondary analysis, the dataset was restricted to an analytic sample of 4,399 AGYW, representing those with complete data on social support, intimate partner violence (IPV) and personal alcohol use. Eligibility required written informed consent (ages 18–24), or assent with guardian consent (under 18), with exclusions for cognitive or communication barriers or unavailability during survey hours. We used baseline cross-sectional data from the HERStory 1 study to examine if/to what extent social support measures are associated with IPV, and to assess the moderating effect of victim alcohol use on the relationship between social support and IPV. An interviewer-administered questionnaire was completed with each participant, in the participant’s language of choice, comprising socio-demographic information including age, marital status, occupation, employment, and educational status; while sections containing sensitive information like sexual behaviours and other reproductive health related questions were self-completed by the participants to ensure privacy and confidentiality, as well as limit socially desirable bias.

### Outcome variable: intimate partner violence

To assess IPV and its forms (emotional, physical and sexual) from an intimate partner (boyfriend or partner) in the previous year, a modified version of the questionnaire from the World Health Organization’s 2005 Multi-Country Study on Women’s Health and Domestic Violence Against Women was utilized (WHO, 2021). This questionnaire has been validated in several settings and populations, ensuring its reliability and applicability across diverse contexts (Badenes-Sastre et al., 2024; Murphy et al., 2024; Oyekunle et al., 2023).

IPV was assessed using a 10-item instrument covering three domains: emotional IPV was measured with three items related to experiences of being insulted, humiliated or threatened by a partner; physical IPV had five items related to slapping, pushing, hitting with a fist or object, kicking/dragging, or threats and use of weapons, while sexual IPV was had two items addressing forced sexual intercourse and coerced sexual acts. Response options for all items were: ‘never’, ‘once’, ‘more than once’, ‘I haven’t had a boyfriend or partner in the last 12 months’, and ‘I prefer not to say’. For analysis, these were recoded into a binary variable, where ‘never’, ‘I haven’t had a boyfriend or partner in the last 12 months’, and ‘I prefer not to say’ were coded as 0, while ‘once’ and ‘more than once’ were coded as 1.

### Main exposure variables

The two main exposure variables were social support and alcohol use by the victim.

#### Social support

The Multidimensional Scale of Perceived Social Support (MSPSS) was used to quantify to what extent AGWY felt supported by their family, friends and significant other (Zimet et al., 1990). The scale’s ability to measure social support from three sources among young people in diverse circumstances has previously been examined (Nearchou et al., 2022; Oon-Arom et al., 2021; Wang et al., 2021). In total, a 12-item scale were used, four for each area of perceived support (family, friends, significant other). Participants rated each item on a 7-point Likert scale, ranging from ‘very strongly disagree’ (WHO, 2021) to ‘very strongly agree’ (Chadambuka & Warria, 2019). Mean scores were calculated for each subscale and for the overall scale. Mean scores between 1.0–2.9 indicated low support, 3.0–5.0 indicated moderate support, and 5.1–7.0 indicated high support. The internal consistency for the overall MSPSS was adequate, with a Cronbach’s alpha of .91, while the Cronbach’s alpha for the sub-scales were .85, .88 and .93 for family, friends and significant other respectively.

#### Alcohol use

Harmful drinking was measured using the Alcohol Use Disorders Identification Test-Consumption (AUDIT-C), a three-item alcohol screening tool designed to identify individuals with risky drinking behaviors (Morojele et al., 2017). This tool is derived from the Alcohol Use Disorders Identification Test (AUDIT), a 10-item screening tool developed by the World Health Organization (Saunders et al., 1993). The AUDIT-C consists of three questions, each scored on a scale from 0 to 4, with a total possible score ranging from 0 to 12. One of the questions asked is, *‘How often do you have a drink containing alcohol?’* The response options are: 0 for Never, 1 for Monthly or less, 2 for 2–4 times a month, 3 for 2–3 times a week, and 4 for 4 or more times a week. A score of 2 or higher indicates a potential risk for hazardous drinking or alcohol use disorder, This tool has been widely used and validated in South African settings, confirming its reliability in assessing harmful drinking behaviors within this context (Goldschmidt et al., 2023). In this study, the AUDIT-C demonstrated strong reliability, with a Cronbach’s alpha coefficient of .92, indicating a high level of internal consistency.

### Data analysis

Firstly, descriptive statistics for the sample population of adolescent girls and young women (AGYW) were undertaken, including frequencies and percentages of socio-demographic characteristics and the distribution of study variables. Second, associations between perceived social support (measured using the MSPSS) and various types of IPV (emotional, physical and sexual IPV) were examined using Pearson chi-square tests. Third, adjusted logistic regression models were fitted to examine the associations between perceived social support and IPV outcomes while adjusting for socio-demographic factors, including age, employment status, education level and relationship status. Fourth, to assess whether alcohol use modified the association between perceived social support and IPV, interaction terms between alcohol use and the different social support domains were included in the regression models. Adjusted Wald tests were used to evaluate the statistical significance of the interaction terms. Predicted probabilities were estimated from the interaction models to facilitate interpretation of the moderation effects. These results are presented as marginal effects in a Table and graphically in the supplementary material All analyses were repeated separately for adolescents (15–19 years) and young adults (20–24 years). Statistical analyses were conducted using STATA 19, accounting for the complex survey design and clustering.

## Results

### Sociodemographic characteristics and outcome variables

A total of 4,399 AGYW aged 15–24 years were included in the analytic sample for this study, with Table 1 summarizing their socio-demographic characteristics and the distribution of other variables. Of the sample, 57.24% were aged 15–19 years, while 42.76% were aged 20–24 years. Regarding work history, most (81.02%) had never worked, with 57.24% currently being in school and 42.76% not, while the relationship status distribution indicates that approximately two-thirds (62.11%) were in a relationship (married or dating). Past 12-month IPV was common, with 28.71% experiencing any IPV, 23.12% emotional IPV, 17% physical IPV and 7% past year sexual IPV experience, while 25.23% reported harmful alcohol use.

### Distribution of perceived social support

Table 2 shows that perceived social support was generally high across all MSPSS domains, with over half of participants reporting high overall support (57.13%). High support was most common for support from significant others (62.76%) and family (62.06%), whereas support from friends had the lowest proportion of high support (48.94%) and the highest proportion of low support (15.84%). Similar patterns were observed among adolescents and young adults.

### Bivariate association between social support, alcohol use, and IPV

Pearson chi-square tests were used to examine associations between perceived social support (measured using the MSPSS) and various types of IPV (emotional, physical, and sexual IPV). The results (Table 3) indicate that all forms of social support were significantly associated with every type of IPV (*p* < 0.001), demonstrating a strong and consistent relationship between higher levels of perceived support and a lower likelihood of experiencing IPV. In the age-stratified analysis, these associations generally remained significant among both adolescents (15–19 years) and young adults (20–24 years). However, some variations were observed among young adults, where the associations between support from significant others and physical IPV, and between family support and emotional IPV, were not statistically significant. Harmful alcohol use remained significantly associated with emotional, physical, and sexual IPV in both age groups.

### Association between social support, alcohol use, and IPV based on multivariable models

Table 4 reports the multivariate analysis, adjusting for socio-demographic variables (age, employment status, education level and relationship status). Overall, higher levels of perceived social support were associated with lower odds of emotional, physical, and sexual IPV across all support domains. Family and friends’ support showed particularly strong protective associations. In the age-stratified analysis, the protective effects of social support were generally stronger and more consistent among adolescents (15–19 years) than young adults (20–24 years). Several associations that remained significant among adolescents were attenuated or no longer significant among young adults, particularly for support from significant others and friends. Harmful alcohol use remained independently associated with increased odds of emotional, physical, and sexual IPV in both age groups.

### Moderating effect of alcohol use on the association between perceived social support and IPV

In Table 5, the marginal effects of perceived social support on emotional, physical, and sexual IPV by alcohol use status is presented. Adjusted Wald tests indicated that alcohol use significantly modified several social supports – IPV associations, particularly for overall and friends’ support in emotional IPV, overall, significant other and friends’ support in physical IPV, and overall and significant other support in sexual IPV. Across all IPV outcomes, predicted probabilities were consistently higher among alcohol users than non-/moderate alcohol users. Similar patterns were observed among adolescents and young adults, although predicted probabilities were generally higher among young adults, indicating a higher likelihood of experiencing IPV across most levels of social support and alcohol use. Graphical presentations of the marginal effects are provided in the supplementary material (sFigures 1–36).

## Discussion

This study examines association between perceived social support, alcohol use and the experience of IPV among AGYW in South Africa. The findings suggest that individuals with higher perceived social support are generally less likely to experience emotional, physical and sexual IPV, reinforcing previous research on the protective role of social support in reducing violence (Richardson et al., 2022; Zavala & Kurtz, 2021). However, the protective effect of social support varies across different sources, with family and friends’ support showing the most consistent protective associations. In addition, harmful alcohol use was associated with increased IPV risk and appeared to weaken several social supports – IPV relationships. Age-stratified analyses further suggested that the protective effects of social support were stronger and more consistent among adolescents than among young adults.

Consistent with a previous study (Oyekunle et al., 2023), emotional IPV was the most commonly reported form, followed by physical and sexual, the findings indicating that higher levels of perceived social support are linked to lower odds of experiencing violence across these domains. Support from friends and family consistently provided the strongest protective benefits, likely due to their role in offering emotional reassurance, tangible assistance and intervention in abusive situations. These results align with prior research showing that strong social networks reduce stress, enhance coping, and increase the likelihood of seeking help or leaving abusive relationships, underscoring the central role of family and peer support in reducing vulnerability to IPV (Ogbe et al., 2020).

The protective effects of social support were not uniform across support sources. Support from a significant other showed a comparatively weaker association with sexual IPV particularly in the context of harmful alcohol use, which may be explained by the complex reality in which the intimate partner is often both the source of violence and the expected source of support. When sexual IPV occurs within a relationship, the partner’s role as a perpetrator fundamentally undermines his role as a supporter, making such ‘support’ ineffective or even contradictory. Survivors may therefore rely more heavily on external sources of support, such as family or friends, which showed more consistent protective associations across IPV types generally, indicating that reliance on a partner for emotional support may not be sufficient to prevent coercive sexual experiences within a relationship. This finding aligns with previous studies which suggest that survivors of sexual IPV often benefit most from external social networks, such as family, peer support and even social media (Osborn et al., 2024; Wood et al., 2021). The comparatively weaker protective role of significant other support against sexual IPV suggests that social support structures external to the relationship are essential for reducing the risk of intimidation and violence.

The age-stratified analysis provides additional insight into how the relationship between perceived social support and IPV differs between adolescents and young adults. Overall, the protective association of social support was more consistent among adolescents (15–19 years) compared to young adults (20–24 years). Among adolescents, higher perceived social support across most support domains remained significantly associated with lower odds of all forms of IPV, suggesting that social support may play a particularly important protective role during this developmental stage (Ragavan et al., 2020). Conversely, among young adults, the associations were less consistent, with some support domains, including support from significant others for physical IPV and family support for emotional IPV no longer showing statistically significant associations. This may reflect shifts in social dynamics that occur during the transition to young adulthood, where reliance on family may decrease and peer or partner relationships become more complex and, at times, less protective (Oliveira et al., 2020). It is also possible that young adults experience different types or contexts of relationships, which may influence how social support functions in relation to IPV risk (Ragavan et al., 2020). The findings for sexual IPV showed relatively more consistency across both age groups, with social support remaining associated across several domains, suggesting that broader social networks, particularly family and friends, may continue to play an important role in reducing vulnerability to sexual IPV regardless of age (Giordano et al., 2020; Makleff et al., 2020). Taken together, these results highlight that while social support is an important protective factor across adolescence and young adulthood, its influence may be stronger and more stable earlier in life course. This underscores the importance of strengthening social support systems during adolescence, while also recognizing that interventions targeting young adults may need to account for changing social structures and relationship dynamics (Mastorci et al., 2024).

A key finding of this study is the moderating role of alcohol use in the relationship between social support and IPV. While high social support generally reduces the risk of IPV, the protective effect appears to be attenuated among individuals who engage in harmful alcohol use, with significant moderation observed across selected social support domains for emotional, physical and sexual forms of abuse. Emotional IPV may escalate where alcohol impairs communication and increases volatility, as reported in previous studies (Hildebrand Karlén et al., 2021; Roach et al., 2022). Similarly, physical IPV is more likely to occur when alcohol contributes to increasing aggression and impulsivity, and lowering inhibitions (Eckhardt et al., 2015). Harmful alcohol use has also been linked to sexual coercion by reducing an individual’s capacity to resist unwanted advances (Shaw & Read, 2021), these dynamics help to explain why it undermines the protective role of social support. Normally, social support buffers against IPV by fostering healthier coping, improving conflict resolution and providing external resources. However, when alcohol use is present, these protective pathways are weakened, as communication within supportive relationships becomes impaired, reliance on social networks is disrupted, and impulsive or aggressive behavior may override the benefits of support (Kirwan et al., 2022). These patterns were observed across both adolescents and young adults, though predicted probabilities of IPV were generally higher among young adults, suggesting greater overall vulnerability in this age group. These findings reinforce prior literature that identifies alcohol use as a serious risk factor for IPV perpetration and victimization (Reese et al., 2021; Spencer & Stith, 2020). The attenuation of social support’s protective role in the presence of alcohol use suggests that interventions should simultaneously target harmful drinking and strengthen social support systems by integrating substance abuse prevention and treatment with IPV prevention and community-based support programs tailored for AGYW.

Despite its contributions, this study has several limitations. The cross-sectional design limits the ability to establish causality between social support and IPV experiences, and the observed relationships may be bidirectional; longitudinal studies may provide better insight into the temporal ordering of these associations. Second, the study relies on self-reported data, which may be subject to recall and social desirability biases. Furthermore, while the study accounts for key demographic variables, unmeasured confounders, such as mental health status, history of childhood abuse, and relationship dynamics, could influence the observed associations. The possibility that unmeasured confounders could affect the results highlights the need for causal inference methods in future studies. In addition, alcohol use was measured using the AUDIT-C, a screening tool that may not fully capture patterns of alcohol consumption and may misclassify non-hazardous drinking. Finally, the data were collected prior to the COVID-19 pandemic and may not fully reflect current patterns of IPV and associated risk factors.

Nonetheless, this study has notable strengths, one being its population-based design, which ensured representativeness of AGYW across multiple districts and enhances the generalizability of the findings. It also contributes to the growing body of evidence on the protective role of social support in IPV prevention, offering insight into how different sources of support interact with IPV risk across emotional, physical, and sexual domains. The inclusion of multiple forms of IPV (emotional, physical, and sexual) offers a comprehensive perspective, emphasizing the complex nature of such experiences and the importance of tailored intervention strategies. Furthermore, the examination of alcohol as a moderating factor underscores the need for integrated approaches that combine social support mechanisms with substance use interventions. The age-stratified analysis, spanning adolescents and young adults, adds further depth by enabling a nuanced understanding of how protective mechanisms operate across developmental stages, thereby informing more targeted prevention and intervention programs for each age group.

## Conclusion

In conclusion, while this study’s findings underscore the protective role of social support in reducing the risk of IPV across its various forms, they highlight that not all sources of support are equally protective, and that harmful alcohol use significantly weakens the buffering effect of social networks. These insights have important implications for IPV prevention and intervention programs, suggesting that efforts should focus on strengthening social support systems while simultaneously addressing alcohol-related risk factors. Future research should explore additional contextual and interpersonal factors that may influence these relationships, with the aim of developing more effective, holistic strategies that address both social and behavioral determinants of IPV risk.

## Supplementary Material

Supplemental data for this article can be accessed online at https://doi.org/10.1080/17450128.2026.2702907

Supplementary Figures

## Figures and Tables

**Table 1:** Sociodemographic characteristics and outcome variables *(N = 4399)*

Variable	Category	n (%)
Age group (years)	15–19	2518 (57.24)
	20–24	1881 (42.76)
Work History	Some work	835 (18.98)
	Never worked	3564 (81.02)
Education Status (Currently in School)	No	1881 (42.76)
	Yes	2518 (57.24)
Relationship Status	Single	1624 (36.92)
	In a relationship (married/dating)	2732 (62.11)
	Other	43 (0.98)
Emotional IPV	No	3382 (76.88)
	Yes	1017 (23.12)
Physical IPV	No	3651 (83.04)
	Yes	748 (17.00)
Sexual IPV	No	4120 (93.66)
	Yes	279 (6.34)
Alcohol	No/moderate use	3289 (74.77)
	Harmful use	1110 (25.23)

**Table 2:** Distribution of perceived social support in the study; *N* = 4399

Multidimensional Scale of Perceived Social Support (MSPSS)				Age-Stratified	
				Adolescent (15–19) *N* = 2518	Young Adults (20–24) *N* = 1881
		n (%)		n (%)	n (%)
Overall Perceived Social Support	Low Perceived Support (1–2.9)	382 (8.68)		204 (8.10)	178 (9.46)
	Medium Perceived Support (3–5)	1504 (34.19)		815 (32.37)	689 (36.63)
	High Perceived Support (5.1–7)	2513 (57.13)		1499 (59.53)	1014 (53.91)
Perceived Social Support (Significant Other Subscale)	Low Perceived Support (1–2.9)	439 (9.98)		244 (9.69)	195 (10.37)
	Medium Perceived Support (3–5)	1199 (27.26)		697 (27.68)	502 (26.69)
	High Perceived Support (5.1–7)	2761 (62.76)		1577 (62.63)	1184 (62.95)
Perceived Social Support (Family Subscale)	Low Perceived Support (1–2.9)	462 (10.50)		253 (10.05)	209 (11.11)
	Medium Perceived Support (3–5)	1207 (24.44)		707 (28.08)	500 (26.58)
	High Perceived Support (5.1–7)	2730 (62.06)		1558 (61.87)	1172 (62.31)
Perceived Social Support (Friends Subscale)	Low Perceived Support (1–2.9)	697 (15.84)		329 (13.07)	368 (19.56)
	Medium Perceived Support (3–5)	1549 (35.21)		852 (33.84)	697 (370.05)
	High Perceived Support (5.1–7)	2153 (48.94)		1337 (53.10)	816 (43.38)

**Table 3:** Bivariate analysis showing the association between perceived social support and intimate partner violence

	Perceived Support		Emotional IPV			(15–19)	(20–24)	Physical IPV			(15–19)	(20–24)	Sexual IPV			(15–19)	(20–24)
			No Emotional IPV	Emotional IPV	P-value			No Physical IPV	Physical IPV	P-value			No Sexual IPV	Sexual IPV	P-value		
	Low		260 (68.06)	122 (31.94)	<0.001	<0.001	<0.05	298 (78.01)	84 (21.99)	<0.001	<0.001	<0.05	344 (90.04)	38 (9.95)	<0.001	<0.001	<0.01
Overall	Medium		1095 (72.81)	409 (27.19)				1193 (79.32)	311 (20.68)				1377 (91.56)	127 (8.44)			
	High		2027 (80.66)	486 (19.34)				2160 (85.95)	353 (140.05)				2399 (95.46)	114 (4.54)			
	Low		317 (72.21)	122 (27.79)	<0.01	<0.001	<0.01	351 (79.95)	88 (20.05)	<0.01	<0.05	0.1477	408 (92.94)	31 (7.06)	<0.001	<0.01	<0.001
Significant other	Medium		889 (74.15)	310 (25.85)				973 (81.15)	226 (18.85)				1094 (91.24)	105 (8.76)			
	High		2176 (78.81)	585 (21.19)				2327 (84.28)	434 (15.72)				2618 (94.82)	143 (5.18)			
	Low		314 (67.97)	148 (32.03)	<0.001	<0.001	0.1558	359 (77.71)	103 (22.29)	<0.001	<0.001	<0.01	413 (89.39)	49 (10.61)	<0.001	<0.001	<0.001
Family	Medium		875 (72.49)	332 (27.51)				948 (78.54)	259 (21.46)				1102 (91.30)	105 (8.70)			
	High		2193 (80.33)	537 (19.67)				2344 (85.86)	386 (14.14)				2605 (95.42)	125 (4.58)			
	Low		459 (65.85)	238 (34.15)	<0.001	<0.001	<0.01	530 (76.04)	167 (23.96)	<0.001	<0.001	0.1675	630 (90.39)	67 (9.61)	<0.001	<0.001	<0.05
Friends	Medium		1180 (76.18)	369 (23.82)				1264 (81.60)	285 (18.40)				1439 (92.90)	110 (7.10)			
	High		1743 (80.96)	410 (19.04)				1857 (86.25)	296 (13.75)				2051 (95.26)	102 (4.74)			
Alcohol use	No/moderate use		2702 (82.15)	587 (17.85)	<0.001	<0.001	<0.001	2869 (87.23)	420 (12.77)	<0.001	<0.001	<0.001	3125 (95.01)	164 (4.99)	<0.001	<0.001	<0.001
	Harmful use		680 (61.26)	430 (38.74)				782 (70.45)	328 (29.55)				995 (89.64)	115 (10.36)			

**Table 4:** Multivariate analysis on the role of perceived social support and alcohol risk again various IPV outcomes

MSPSS Category	Support category	Emotional IPV							Physical IPV							Sexual IPV					
		OR	95% Cl	aOR	95% Cl	aOR by Age group			OR	95% Cl	aOR	95% Cl	aOR by Age group				95% Cl	aOR	95% Cl	aOR by Age group	
						(Aboagye et al., 2023; Howell et al., 2018; Ryan et al., 2018; Sere et al., 2021; Thaggard and Montayre, 2019)	(Badenes-Sastre et al., 2024; Davies et al., 2023; Mathews et al., 2020; Murphy et al., 2024; Oyekunle et al., 2023)						(Aboagye et al., 2023; Howell et al., 2018; Ryan et al., 2018; Sere et al., 2021; Thaggard and Montayre, 2019)	(Badenes-Sastre et al., 2024; Davies et al., 2023; Mathews et al., 2020; Murphy et al., 2024; Oyekunle et al., 2023)		OR				(Aboagye et al., 2023; Howell et al., 2018; Ryan et al., 2018; Sere et al., 2021; Thaggard and Montayre, 2019)	(Badenes-Sastre et al., 2024; Davies et al., 2023; Mathews et al., 2020; Murphy et al., 2024; Oyekunle et al., 2023)
Overall Scale	Low Perceived Support Medium Perceived Support	0.80	0.62, 1.01	0.78	0.60, 1.00	0.69**	0.90		0.92	0.71, 1.21	0.91	0.68, 1.20	0.78	1.08		0.83	0.57, 1.22	0.83	0.56, 1.23	0.50**	1.40
	High Perceived Support	0.51**	0.40, 0.65	0.51**	0.40, 0.65	0.38**	0.71**		0.58**	0.44, 0.76	0.58**	0.44, 0.77	0.43**	0.80		0.43**	0.29, 0.63	0.44**	0.30, 0.65	0.24**	0.82
Significant other Subscale	Low Perceived Support Medium Perceived Support	0.91	0.71, 1.16	0.92	0.72, 1.19	0.68**	1.25		0.93	0.70, 1.22	0.94	0.71, 1.24	0.82	1.07		1.26	0.83, 1.92	1.31	0.86, 2.00	0.75	2.21**
	High Perceived Support	0.70**	0.56, 0.88	0.68**	0.54, 0.86	0.56**	0.86		0.74**	0.58, 0.96	0.72**	0.56, 0.94	0.64**	0.83		0.72	0.48, 1.07	0.72	0.48, 1.08	0.47**	1.11
Family Subscale	Low Perceived Support																				
	Medium Perceived Support	0.81	0.64, 1.02	0.81	0.64, 1.04	0.83	0.79		0.95	0.74, 1.23	0.97	0.74, 1.27	0.93	1.02		0.80	0.56, 1.15	0.82	0.57, 1.18	0.58**	1.10
Friends Subscale	High Perceived Support Low Perceived Support	0.52**	0.42, 0.65	0.53**	0.42, 0.66	0.38**	0.74		0.57**	0.45, 0.73	0.59**	0.45, 0.75	0.49**	0.70**		0.40**	0.29, 0.57	0.41**	0.29, 0.58	0.30**	0.54**
	Medium Perceived Support	0.60**	0.50, 0.73	0.63**	0.51, 0.77	0.53**	0.71**		0.72**	0.58, 0.89	0.75**	0.60, 0.94	0.57**	0.93		0.72**	0.52, 0.99	0.76	0.55, 1.06	0.33**	1.48
	High Perceived Support	0.45**	0.38, 0.55	0.49**	0.40, 0.59	0.35**	0.67**		0.51**	0.41, 0.63	0.55**	0.44, 0.68	0.38**	0.77		0.47**	0.34, 0.64	0.52**	0.37, 0.72	0.25**	0.99
Alcohol use	No/moderate Alcohol use Harmful Alcohol use	2.91**	2.50, 3.38	2.56**	2.19, 2.99	2.49**	2.53**		2.87**	2.43, 3.38	2.52**	2.13, 2.99	2.53**	2.45**		2.20**	1.72, 2.82	1.93**	1.50, 2.50	1.85**	1.95

Reference category: Low Perceived Support / No/moderate Alcohol use.

** *p* < 0.05.

**Table 5:** Marginal effects of Perceived social support on IPV by alcohol use status and significant interaction effects

IPV	MSPSS	Margins, 95% CI (Alcohol -)	Margins, 95% CI (Alcohol +)	Margins, 95% CI (Alcohol -)	Margins, 95% CI (Alcohol +)	Margins, 95% CI (Alcohol -)	Margins, 95% CI (Alcohol +)
				Adolescents (15 – 19)		Young Adults (20 – 24)	
Emotional	Overall^†‡^	0.13 (0.12, 0.15)	0.37 (0.33, 0.40)	0.10 (0.08, 0.11)	0.29 (0.25, 0.34)	0.19 (0.17, 0.22)	0.44 (0.39, 0.50)
	Significant other	0.15 (0.14, 0.17)	0.38 (0.34, 0.41)	0.12 (010, 0.14)	0.32 (0.28, 0.37)	0.20 (0.17, 0.23)	0.43 (0.38, 0.49)
	Family^‡^	0.14 (0.13, 0.16)	0.36 (0.32, 0.40)	0.09 (0.08, 0.11)	0.28 (0.23, 0.33)	0.22 (0.19, 0.25)	0.44 (0.38, 0.49)
	Friends^†‡^	0.13 (0.11, 0.15)	0.37 (0.33, 0.41)	0.09 (0.08, 0.11)	0.30 (0.24, 0.35)	0.19 (0.16, 0.23)	0.45 (0.39, 0.51)
Physical	Overall^†^	0.09 (0.08, 0.11)	0.28 (0.24, 0.31)	0.07 (0.05, 0.08)	0.22 (0.18, 0.27)	0.14 (0.11, 0.16)	0.33 (0.28, 0.38)
	Significant other^†^	0.11 (0.10, 0.12)	0.29 (0.26, 0.32)	0.08 (0.07, 0.10)	0.24 (0.20, 0.29)	0.14 (0.12, 0.17)	0.34 (0.29, 0.39)
	Family	0.10 (0.09, 0.12)	0.26 (0.22, 0.29)	0.08 (0.06, 0.09)	0.21 (0.16, 0.25)	0.15 (0.12, 0.17)	0.30 (0.26, 0.35)
	Friends^†§^	0.09 (0.08, 0.10)	0.28 (0.24, 0.32)	0.07 (0.05, 0.08)	0.21 (0.16, 0.26)	0.13 (0.10, 0.15)	0.36 (0.30, 0.41)
Sexual	Overall^†‡^	0.03 (0.02, 0.04)	0.09 (0.07, 0.11)	0.02 (0.01, 0.03)	0.07 (0.04, 0.09)	0.05 (0.03, 0.06)	0.12 (0.08, 0.15)
	Significant other^†§^	0.03 (0.03, 0.04)	0.10 (0.08, 0.12)	0.03 (0.02, 0.04)	0.07 (0.04, 0.10)	0.05 (0.03, 0.06)	0.13 (0.09, 0.16)
	Family^‡^	0.03 (0.02, 0.04)	0.09 (0.07, 0.11)	0.02 (0.01, 0.03)	0.07 (0.04, 0.10)	0.05 (0.03, 0.06)	0.10 (0.07, 0.13)
	Friends	0.03 (0.02, 0.04)	0.09 (0.07, 0.11)	0.02 (0.01, 0.03)	0.06 (0.03,0.09)	0.05 (0.03, 0.07)	0.13 (0.08, 0.17)

† Significant interaction between perceived social support and alcohol use in the overall sample (Adjusted Wald test, *p* < 0.05).

‡ Significant interaction between perceived social support and alcohol use among adolescents aged 15–19 years (Adjusted Wald test, *p* < 0.05).

§ Significant interaction between perceived social support and alcohol use among young adults aged 20–24 years (Adjusted Wald test, *p* < 0.05).

## Data Availability

The data that support the findings of this study are not publicly available due to ethical and legal restrictions to protect participant confidentiality. In accordance with the conditions of the study’s ethical approval, data access is restricted to the research team. Researchers interested in further information about the data may contact the corresponding author, subject to ethical approval and institutional requirements.

## References

[R1] Aboagye RG, Seidu A-A, Cadri A, Salihu T, Arthur-Holmes F, Sam ST, Ahinkorah BO (2023). Ending violence against women: Help-seeking behaviour of women exposed to intimate partner violence in sub-saharan Africa. PLOS ONE.

[R2] Badenes-Sastre M, Spencer CM, Alonso-Ferres M, Lorente M, Expósito F (2024). How severity of intimate partner violence is perceived and related to attitudinal variables? A systematic review and meta-analysis. Aggression & Violent Behavior.

[R3] Camp AR (2022). From experiencing abuse to seeking protection: Examining the shame of intimate partner violence. UC Irvine L Rev.

[R4] Chadambuka C, Warria A (2019). Hurt or help? Understanding intimate partner violence in the context of social norms as practised in rural areas. Social Work/Maatskaplike Werk.

[R5] Conner DH (2013). Financial freedom: Women, money, and domestic abuse. William & Mary Journal of Race, Gender, and Social Justice.

[R6] Davies RL, Rice K, Rock AJ (2023). A systematic review of informal supporters of intimate partner violence survivors: The intimate partner violence model of informal supporter readiness. PeerJ.

[R7] Eckhardt CI, Parrott DJ, Sprunger JG (2015). Mechanisms of alcohol-facilitated intimate partner violence. Violence Against Women.

[R8] Giordano PC, Copp JE, Manning WD, Longmore MA (2020). When worlds collide: Linking involvement with friends and intimate partner violence in young adulthood. Social Forces.

[R9] Goldschmidt L, Mncina B, Langa M, Rebello S, Budaza T, Tshabalala J, Kinfu Y, Achoki T (2023). AUDIT-C as a possible source of referral during the COVID-19 pandemic for participants presenting patterns of high-risk alcohol consumption in a South African township. BMC Public Health.

[R10] Hildebrand Karlén M, Green J, Larsson A, Gudjonsson GH (2021). Time and alcohol do not change everything: How intoxicated witnesses perceive aggression in intimate partner violence. Journal of Interpersonal Violence.

[R11] Hing N, O’mullan C, Mainey L, Nuske E, Breen H, Taylor A (2021). Impacts of male intimate partner violence on women: A life course perspective. International Journal of Environmental Research and Public Health.

[R12] Howell KH, Thurston IB, Schwartz LE, Jamison LE, Hasselle AJ (2018). Protective factors associated with resilience in women exposed to intimate partner violence. Psychology of Violence.

[R13] Kirwan M, Davis KC, Stappenbeck CA, George WH (2022). The roles of emotion regulation, alcohol consumption, and women’s condom request style in men’s coercive condom use resistance intentions. Journal of Aggression, Maltreatment, & Trauma.

[R14] Machisa MT, Christofides N, Jewkes R (2018). Social support factors associated with psychological resilience among women survivors of intimate partner violence in Gauteng, South Africa. Global Health Action.

[R15] Makleff S, Garduño J, Zavala RI, Barindelli F, Valades J, Billowitz M, Silva Márquez VI, Marston C (2020). Preventing intimate partner violence among young people-a qualitative study examining the role of comprehensive sexuality education. Sexuality Research & Social Policy.

[R16] Mastorci F, Lazzeri MFL, Vassalle C, Pingitore A (2024). The transition from childhood to adolescence: Between health and vulnerability. Children.

[R17] Mathews CL, Carl P, Adrian C, Mireille A, Kassahun J, Ncebakazi K, Kuo C, Lovette A, Beauclair R, Cawood C, Khanyile D (2020). Evaluation of a South African Combination HIV Prevention Programme for Adolescent Girls and Young Women: Herstory Study.

[R18] Morojele NK, Nkosi S, Kekwaletswe CT, Shuper PA, Manda SO, Myers B, Parry CD (2017). Utility of brief versions of the alcohol use disorders identification test (AUDIT) to identify excessive drinking among patients in HIV care in South Africa. Journal of Studies on Alcohol and Drugs.

[R19] Murphy M, Smith ER, Chandarana S, Ellsberg M (2024). Experience of intimate partner violence and non-partner sexual violence in conflict-affected settings: A systematic review and meta-analysis. Trauma, Violence, & Abuse.

[R20] Nearchou F, Davies A, Hennessy E (2022). Psychometric evaluation of the multi-dimensional scale of Perceived social support in young adults with chronic health conditions. Irish Journal of Psychological Medicine.

[R21] Ogbe E, Harmon S, Van den Bergh R, Degomme O (2020). A systematic review of intimate partner violence interventions focused on improving social support and/mental health outcomes of survivors. PLOS ONE.

[R22] Oliveira C, Fonseca G, Sotero L, Crespo C, Relvas AP (2020). Family dynamics during emerging adulthood: Reviewing, integrating, and challenging the field. Journal of Family Theory & Review.

[R23] Oon-Arom A, Wongpakaran T, Kuntawong P, Wongpakaran N (2021). Attachment anxiety, depression, and perceived social support: A moderated mediation model of suicide ideation among the elderly. International Psychogeriatrics.

[R24] Osborn M, Ball T, Rajah V (2024). Peer support work in the context of intimate partner violence: A scoping review. Trauma, Violence, & Abuse.

[R25] Oyekunle V, Gibbs A, Tomita A (2023). Assessing the role of depression in reducing intimate partner violence perpetration among young men living in urban informal settlements using a mediation analysis of the stepping stones and creating futures intervention. Global Health Action.

[R26] Pakhomova TE, Dietrich JJ, Closson K, Smit J, Hornschuh S, Smith P, Beksinska M, Ndung’u T, Brockman M, Gray G, Kaida A (2021). Intimate partner violence, depression, and anxiety are associated with higher perceived stress among both young men and women in Soweto and Durban, South Africa. Frontiers in Reproductive Health.

[R27] Ragavan MI, Culyba AJ, Shaw D, Miller E (2020). Social support, exposure to parental intimate partner violence, and relationship abuse among marginalized youth. Journal of Adolescent Health.

[R28] Reese BM, Chen MS, Nekkanti M, Mulawa MI (2021). Prevalence and risk factors of women’s past-year physical IPV perpetration and victimization in Tanzania. Journal of Interpersonal Violence.

[R29] Richardson RA, Haight SC, Hagaman A, Sikander S, Maselko J, Bates LM (2022). Social support and intimate partner violence in rural Pakistan: A longitudinal investigation of the bi-directional relationship. SSM-Population Health.

[R30] Roach AL, Ermer AE, Coleman M, Ganong L (2022). Attitudes about intimate partner violence and alcohol consumption. Journal of Interpersonal Violence.

[R31] Ryan J, Esau MV, Roman NV (2018). Legislative response to family violence in South Africa: A family centered perspective. Aggression & Violent Behavior.

[R32] Sardinha L, Maheu-Giroux M, Stöckl H, Meyer SR, García-Moreno C (2022). Global, regional, and national prevalence estimates of physical or sexual, or both, intimate partner violence against women in 2018. Lancet.

[R33] Saunders JB, Aasland OG, Babor TF, De la Fuente JR, Grant M (1993). Development of the alcohol use disorders identification test (AUDIT): WHO collaborative project on early detection of persons with harmful alcohol consumption-II. Addiction.

[R34] Sere Y, Roman NV, Ruiter RA (2021). Coping with the experiences of intimate partner violence among South African women: Systematic review and meta-synthesis. Frontiers in Psychiatry.

[R35] Sharafudeen S (2024). Lived experience of women facing intimate partner violence.

[R36] Shaw R, Read JP (2021). The differential effects of verbal sexual coercion and forcible sexual assault on alcohol use and consequence trajectories in the first year of college. Psychological Trauma: Theory, Research, Practice, and Policy.

[R37] Spencer CM, Stith SM (2020). Risk factors for male perpetration and female victimization of intimate partner homicide: A meta-analysis. Trauma, Violence, & Abuse.

[R38] Stöckl H, March L, Pallitto C, Garcia-Moreno C (2014). Intimate partner violence among adolescents and young women: Prevalence and associated factors in nine countries: A cross-sectional study. BMC Public Health.

[R39] Thaggard S, Montayre J (2019). Women’s studies international forum.

[R40] Tshoane SM, Bello PO, Mofokeng JT, Olutola AA (2023). Exploration of the gaps in the enactment and implementation of the domestic violence act of South Africa. Khazanah Hukum.

[R41] Wado YD, Mutua MK, Mohiddin A, Ijadunola MY, Faye C, Coll CV, Barros AJD, Kabiru CW (2021). Intimate partner violence against adolescents and young women in sub-saharan Africa: Who is most vulnerable?. Reproductive Health.

[R42] Wang D, Zhu F, Xi S, Niu L, Tebes JK, Xiao S, Yu Y (2021). Psychometric properties of the Multidimensional scale of Perceived social support (MSPSS) among family caregivers of people with schizophrenia in China. Psychology Research and Behavior Management.

[R43] Wood L, Baumler E, Schrag RV, Guillot-Wright S, Hairston D, Temple J, Torres E (2021). “Don’t know where to go for help”: Safety and economic needs among violence survivors during the COVID-19 pandemic. Journal of Family Violence.

[R44] World Health Organization (2021). Violence against women prevalence estimates, 2018: Global, regional and national prevalence estimates for intimate partner violence against women and global and regional prevalence estimates for non-partner sexual violence against women.

[R45] Zavala E, Kurtz DL (2021). Applying differential coercion and social support theory to intimate partner violence. Journal of Interpersonal Violence.

[R46] Zimet GD, Powell SS, Farley GK, Werkman S, Berkoff KA (1990). Psychometric characteristics of the multidimensional scale of perceived social support. Journal of Personality Assessment.

