# Supplementary material for: Examining the influence of alcohol consumption on the protective function of social support in reducing intimate partner violence among adolescent girls and young women
Source: Vulnerable Child Youth Stud. Author manuscript; Available in PMC 2026 Aug 7. (PMC7619332; doi:10.1080/17450128.2026.2702907)
Supplement: Supplementary file 1 [file EMS217086-supplement-Supplementary_Figures.docx]

**Marginal effects plots showing differences in different forms of IPV risk across social support levels for (non-users/moderate users = 0) and (harmful alcohol users = 1) (Fig 1-36).**

Fig 1: This plot illustrates the adjusted probability of experiencing emotional IPV based on overall perceived social support. Individuals with higher perceived support have a lower probability of emotional IPV. However, harmful alcohol use increases the likelihood of emotional IPV across all levels of support, with the highest probability observed among those with low support.

Fig 2: This shows the adjusted predictions of emotional IPV based on social support from significant others. The probability of emotional IPV is lowest among non/reasonable-drinkers with high perceived social support and highest among harmful drinkers with low social support, which suggest that while social support can reduce the risk of emotional IPV, harmful alcohol consumption use may diminish this protective effect.

Fig 3: This plot examines the relationship between family support and emotional IPV. Higher family support is associated with a lower probability of emotional IPV. Harmful alcohol consumption raises the risk of emotional IPV across all support levels, with those having low support experiencing the highest probability.

Fig 4: This plot focuses on social support from friends and emotional IPV. Higher social support from friends is linked to a lower probability of emotional IPV. Harmful alcohol use leads to an increased risk of emotional IPV, with the most pronounced effect among those with low support.

Fig 5: The trend shown in the graph represents the adjusted predictions of physical IPV based on total perceived social support. While higher social support is associated with a lower probability of physical IPV, harmful alcohol use increases the likelihood of physical IPV across all support levels, with the most significant rise for those with medium support.

Fig 6: This figure shows the adjusted predictions of physical IPV based on social support from significant others. The protective effect of support weakens when harmful alcohol is present, with the probability of physical IPV rising sharply for those with medium support.

Fig 7: This visual indicates that the adjusted predictions of physical IPV based on perceived social support from family. While higher family support is associated with a lower probability of physical IPV, harmful alcohol use significantly increases the likelihood of IPV, particularly for those with medium support.

Fig 8: This plot illustrates the effects of social support from friends. Although greater support from friends is linked to a lower probability of physical IPV, harmful alcohol use still elevates physical IPV risk across all levels of support, with the highest probability observed among those with low support.

Fig 9: The plot above presents the adjusted predictions of sexual IPV based on total perceived social support. While higher social support is linked to a lower probability of experiencing sexual IPV, harmful alcohol use increases the risk across all levels of support, with the gap between support levels narrowing when alcohol is involved.

Fig 10: This trend focuses on social support from a significant other. Medium support appears to be associated with the highest increase in sexual IPV risk when harmful alcohol consumption is present, while those with high support experience the lowest probability of sexual IPV, even with harmful alcohol consumption.

Fig 11: The plotted data above shows the adjusted probability of experiencing sexual IPV based on social support from family. Higher family support is linked to a lower probability of sexual IPV, but harmful alcohol consumption increases the risk across all support levels, with the most significant rise among those with medium support.

Fig 12: This plot presents the relationship between social support from friends and sexual IPV. The probability of experiencing sexual IPV is lowest for those with high support. Harmful alcohol use increases the risk of sexual IPV, but the effect appears consistent across all levels of support.

**Marginal effects plots showing differences in different forms of IPV risk across social support levels for (non-users/moderate users = 0) and (harmful alcohol users = 1) among adolescents (Fig 13-24).**

Fig 13: This plot shows the adjusted probability of emotional IPV based on overall perceived social support among adolescents. Higher support is associated with lower IPV risk. However, harmful alcohol use increases the probability across all support levels, with the highest risk among those with low support.

Fig 14: This figure presents emotional IPV by support from significant others. Adolescents with higher support have lower risk, but harmful alcohol use raises the probability across all groups, reducing the protective effect of support.

Fig 15: This plot illustrates emotional IPV in relation to family support. Higher family support is associated with lower IPV risk, although harmful alcohol use increases the probability across all levels, particularly among those with low support.

Fig 16: This figure shows emotional IPV by support from friends. Higher support is linked to lower IPV risk, but harmful alcohol use increases the likelihood across all levels of support.

Fig 17: This plot presents physical IPV by overall perceived social support. Higher support is associated with lower risk, while harmful alcohol use increases physical IPV across all levels, with a more noticeable increase among those with low and medium support.

Fig 18: This figure shows physical IPV by support from significant others. The protective effect of support is evident, but weakened in the presence of harmful alcohol use.

Fig 19: This plot illustrates physical IPV by family support. Higher support is associated with lower risk, although alcohol use increases IPV probability across all levels.

Fig 20: This figure presents physical IPV by friends’ support. While higher support reduces IPV risk, harmful alcohol use increases the probability across all groups.

Fig 21: This plot shows sexual IPV by overall perceived social support. Differences across support levels are smaller, but harmful alcohol use increases the probability of sexual IPV across all levels.

Fig 22: This figure presents sexual IPV by support from significant others. The pattern is less distinct, with smaller differences between support levels, although alcohol use still increases risk.

Fig 23: This plot illustrates sexual IPV by family support. Higher support is associated with slightly lower risk, but harmful alcohol use raises the probability across all groups.

Fig 24: This figure shows sexual IPV by support from friends. Differences across support levels are modest, and alcohol use consistently increases the likelihood of sexual IPV.

**Marginal effects plots showing differences in different forms of IPV risk across social support levels for (non-users/moderate users = 0) and (harmful alcohol users = 1) among young adults (Fig 25-36).**

Fig 25: This plot shows emotional IPV by overall perceived social support among young adults. Higher support is associated with lower IPV risk, but the overall probabilities are higher than among adolescents, particularly for harmful alcohol users.

Fig 26: This figure presents emotional IPV by support from significant others. While higher support is associated with lower risk, harmful alcohol use substantially increases IPV probability across all levels.

Fig 27: This plot illustrates emotional IPV by family support. The protective effect of support is present but less pronounced, with higher probabilities observed among alcohol users.

Fig 28: This figure shows emotional IPV by friends’ support. Although higher support is associated with lower IPV risk, the differences across levels are smaller compared to adolescents.

Fig 29: This plot presents physical IPV by overall perceived social support. Higher support remains protective, but harmful alcohol use increases IPV probability across all levels.

Fig 30: This figure shows physical IPV by support from significant others. The protective effect is weaker, with less distinction between support levels.

Fig 31: This plot illustrates physical IPV by family support. While higher support is associated with lower risk, alcohol use increases IPV probability, and differences across levels are modest.

Fig 32: This figure presents physical IPV by friends’ support. The pattern is less structured, with smaller differences between support levels.

Fig 33: This plot shows sexual IPV by overall perceived social support. Differences across support levels are small, and harmful alcohol use increases the probability across all groups.

Fig 34: This figure presents sexual IPV by support from significant others. The pattern is inconsistent, with minimal differences between support levels and some overlap.

Fig 35: This plot illustrates sexual IPV by family support. Higher support shows only slight reductions in IPV probability, with alcohol use increasing risk across all levels.

Fig 36: This figure shows sexual IPV by friends’ support. Differences across support levels are minimal, and harmful alcohol use consistently increases IPV risk.
